# On the Employment of Mercury and the Iodide of Potassium in the Treatment of Syphilis

**Published:** 1863-05

**Authors:** 


ON THE EMPLOYMENT OF

### MERCURY AND THE IODIDE OF POTASSIUM IN THE TREATMENT OF SYPHILIS.

By Dr. JUNIEN-LAVILLAUROY.

Having sketched in a bold style the history of the treatment of syphilis by mercury, having pointed out at what period of the disease, and against what symptoms mercurials may be useful, M. Junien-Lavillauroy sums up in his thesis the principal modes of the administration of mercury, and insists specially on the method of Montpellier, or that of radical cure. The first part ends with a table of the principal mercurial compounds, arranged in the order of their activity.

We know that M. Bouchardat divides the principal compounds of mercury into: 1st. *The soluble preparations.* Iodhydrargyrate of the iodide of potassium; bi-chloride (corrosive sublimate); and coanide of mercury.

2d. *Insoluble preparations.* The red oxide; the protochloride; proto-iodide; and metallic mercury.

This arrangement is admitted by all therapeutists, particularly by M. Trousseau.

The second part of this work is devoted to iodide of potassium. The opinions advanced by the author are summed up in the following conclusions:

I. If, with the generality of physicians, we adopt the method of radical cure, or that of Montpellier, in the treatment of constitutional syphilis, the practice which consists in giving the iodide of potassium after the disappearance of the symptoms under mercurial treatment, is always useless and may often become injurious.

II. The mixed treatment is very energetic, and ought to be employed in the case of very obstinate and inveterate syphilis; but with the condition, that, if we wish for a durable cure, the mixed treatment must be continued a long time, or that after it the ordinary mercurial treatment should be continued for a certain period.

III. We understand the prompt and immediate treatment on the part of those physicians who do not believe in the preventive action of what they term anti-syphilitics; but from that moment they give them to anticipate new symptoms, according to the rules of the method of Montpellier (and, it must be remarked, all those who give the iodide of potassium after mercury, do it for this end), all these do the opposite to what they propose to do. They drive, in fact, the mercury from the economy, the specific which they have designedly accumulated in it, and to which they are proposing to add, perhaps injuriously, another specific, the iodide of potassium.

IV. The action of potassium on the compounds of mercury fixed in the economy explains the opinion that iodide of potassium does not act as an anti-syyhilitic in tertiary syphilis, except in those persons who have previously undergone a mercurial treatment.

V. The iodide of potassium may act not so much as iodide of potassium, as in transforming the mercury in the system into a more active compound, the iodhydrargyrate of iodide of potassium. So that there would seem in reality but one specific for syphilis, *mercury*.

VI. When in syphilitic patients symtoms appear a long time after mercurial treatment (and with much greater reason when it has not been employed at all), as we may suppose that there is no mercury left in the system, if we wish to treat the patient by iodide of potassium, in order to count upon its action, it is well to give at the same time a mercurial preparation; that is to say, to follow what Vidal de Cassis calls the mixed treatment.—*Boston Medical and Surgical Journal.*

